# Analysis of novel hyperosmotic shock response suggests ‘beads in liquid’ cytosol structure

**DOI:** 10.1242/bio.044529

**Published:** 2019-07-08

**Authors:** Alexander I. Alexandrov, Erika V. Grosfeld, Alexander A. Dergalev, Vitaly V. Kushnirov, Roman N. Chuprov-Netochin, Pyotr A. Tyurin-Kuzmin, Igor I. Kireev, Michael D. Ter-Avanesyan, Sergey V. Leonov, Michael O. Agaphonov

**Affiliations:** 1 Federal Research Center “Fundamentals of Biotechnology” of the Russian Academy of Sciences, Bach Institute of Biochemistry, Leninsky Ave. 33, bld. 2, Moscow 119071, Russia; 2 A.N. Belozersky Institute of Physico-chemical Biology, M.V. Lomonosov Moscow State University, Leninskie gori 1, bldg 40, Moscow 119234, Russia; 3 Chair of Molecular and Cell Biology, Moscow Institute of Physics and Technology (State University), Institutskiy per. 9, Dolgoprudny, Moscow Region 141701, Russia; 4 School of Biological and Medical Physics, Moscow Institute of Physics and Technology (State University), Institutskiy per. 9, Dolgoprudny, Moscow Region 141701, Russia; 5 Department of Biochemistry and Molecular Medicine, Faculty of Medicine, M.V. Lomonosov Moscow State University, Lomonosovskiy pr., 27 bldg 1, Moscow 119192, Russia; 6 V. I. Kulakov National Medical Research Center for Obstetrics, Gynecology, and Perinatology, Moscow 117198, Russia; 7 Faculty of Biology, M. V. Lomonosov Moscow State University, Moscow 119234, Russia; 8 Institute of Cell Biophysics of the Russian Academy of Sciences, Institutskaya str., 3, Moscow Region, 142290 Puschino, Russia

**Keywords:** Aggregation, Amyloid, Chaperone, Cytoplasm, Foci, Hyperosmotic shock, Liquid–liquid phase separation, P-bodies, Yeast

## Abstract

Proteins can aggregate in response to stresses, including hyperosmotic shock. Formation and disassembly of aggregates is a relatively slow process. We describe a novel instant response of the cell to hyperosmosis, during which chaperones and other proteins form numerous foci with properties uncharacteristic of classical aggregates. These foci appeared/disappeared seconds after shock onset/removal, in close correlation with cell volume changes. Genome-wide and targeted testing revealed chaperones, metabolic enzymes, P-body components and amyloidogenic proteins in the foci. Most of these proteins can form large assemblies and for some, the assembled state was pre-requisite for participation in foci. A genome-wide screen failed to identify genes whose absence prevented foci participation by Hsp70. Shapes of and interconnections between foci, revealed by super-resolution microscopy, indicated that the foci were compressed between other entities. Based on our findings, we suggest a new model of cytosol architecture as a collection of numerous gel-like regions suspended in a liquid network. This network is reduced in volume in response to hyperosmosis and forms small pockets between the gel-like regions.

## Abstract (precis)

**Summary:** We describe a novel cellular response to hyperosmotic shock – rapid reversible formation of foci by various proteins. Data on foci behavior suggest a novel ‘beads in liquid’ cytosolic structure.

## INTRODUCTION

Systems biology aspires to understand life in quantitative detail, eventually allowing complete modeling of living cells *in silico*. For such an endeavor, it is crucial to predict distribution and local concentrations of substances and molecular assemblies within a cell under varying conditions. However, even for simple cytosolic processes, this has been difficult to achieve due to various aspects of cytoplasmic heterogeneity [for a review, see Luby-Phelps (1999, 2013] and differing views on the nature of the cytosol, i.e. is it a simple solution, crowded liquid or a hydrogel (Grygorczyk et al., 2015)? While all of these models describe some properties of the cytosol, they are difficult to unite in a single framework and thus there is a lack of comprehensible mechanistic models. Notably, the cytoskeleton, which accomplishes most of the active transport in the cell, has been implicated in cytosolic structuring (Hu et al., 2017; Provance et al., 1993), however, it does not seem to fully account for existing observations (Weiss et al., 2004). Another difficulty that adds to the complexity of cytosolic structure is the ability of the cytosol to change its viscosity in a dramatic manner, such as during changes of pH (Munder et al., 2016; Parry et al., 2014) or osmotic pressure (Miermont et al., 2013).

Hyperosmotic shock is a ubiquitous environmental factor commonly encountered by microorganisms and multicellular organisms. It is also relevant for some tissues in mammals (Brocker et al., 2012). The response of cells to hyperosmosis has been studied in great detail in terms of the sensing of hyperosmotic shock, the signaling cascades which mediate the cells' responses, and the mechanisms of adaptation to hyperosmotic conditions via synthesis and retention of osmolytes, namely glycerol, in yeast [reviewed in Saito and Posas (2012)]. However, the immediate consequences of hyperosmotic shock are less well characterized.

During hyperosmotic shock, cells shrink due to water efflux. This is accompanied by increased cytoplasmic viscosity and reduction of diffusion rates for various proteins (Miermont et al., 2013), as well as aggregation of cellular proteins and model amyloidogenic proteins in *Caenorhabditis* *elegans* (Burkewitz et al., 2011) and yeast (Han and Emr, 2011; Oeser et al., 2016). Also, in yeast, hyperosmotic shock can influence the disappearance and appearance of prion amyloids (Newnam et al., 2011; Tyedmers et al., 2008). Notably, protein aggregation as well as formation of visible foci in response to various stresses takes a noticeable amount of time, i.e. at least several minutes for severe heat shock. Dissolution of aggregates and foci is an even slower process which can take up to 1 h or more (Wallace et al., 2015).

Another class of entities that can appear in response to changing conditions are protein droplets that form due to liquid–liquid phase separation (LLPS) [reviewed in Hyman et al. (2014); Shin and Brangwynne (2017)]. LLPS can proceed in a matter of seconds in some cases, for instance, when proteins prone to aggregation are rapidly brought into close proximity (Bracha et al., 2018). Notably, a recent study reported that hyperosmosis caused formation of phase-separated droplets in mammalian cells (Cai et al., 2018 preprint).

Gathering of a particular protein into aggregates or other types of assemblies can be monitored *in vivo* by labeling with a fluorescent moiety e.g. green fluorescent protein (GFP). Here we observed that some proteins fused to GFP rapidly formed intracellular foci in response to hyperosmotic shock (OSF, osmotic shock foci). The shape and dynamics of appearance and disappearance of OSFs were inconsistent with classic protein aggregation, but indicated highly reversible formation of entities with unusual properties and morphology. This led us to propose a new model of the cytosol as gel-like ‘beads’ suspended in a liquid network.

## RESULTS

### Chaperones can form cytoplasmic foci in response to hyperosmosis

**Fig. 1.**
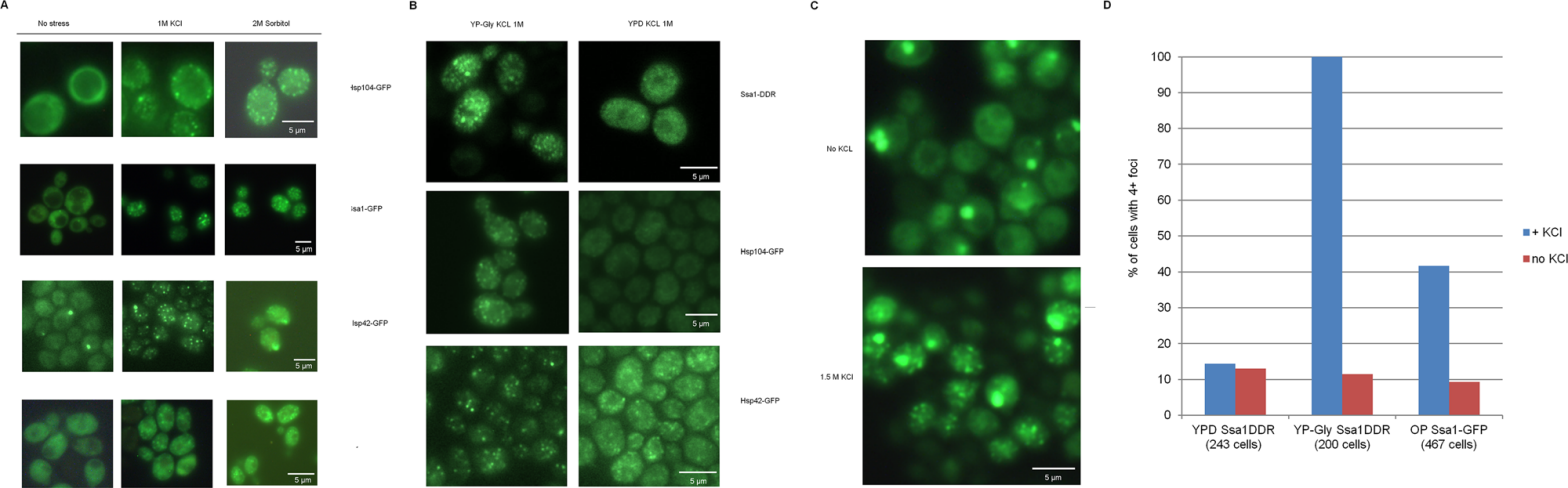
**Chaperones can form OSFs under various stress conditions both in *S. cerevisiae* and *O. parapolymorpha*.** (A) Cells of the BY4741 strain bearing the indicated GFP-fusion protein were grown in YP-Gly medium and then transferred onto medium with the indicated concentration of KCl, glycerol or sorbitol. (B) Cells bearing the indicated GFP-fusion chaperone were grown in YP-Gly or YPD medium to logarithmic phase and transferred onto the same medium supplemented with 1M KCl. (C) Cells of *O. parapolymorpha*, producing the closest Ssa1 homologue tagged with GFP (see Materials and Methods) were grown in YP-Gly medium to logarithmic phase and transferred onto YPD medium with 1M KCl. (D) Analysis of foci numbers in populations of cells used in B and C, with the vertical axis depicting the percentage of cells containing more than three foci. OP, *O. parapolymorpha* cells. Scale bars: 5 µm.

Earlier it was observed that hyperosmotic shock causes aggregation of cellular proteins and model amyloidogenic proteins in *C. elegans* (Burkewitz et al., 2011), as well as influences disappearance and appearance of prion amyloids in yeast (Newnam et al., 2011; Tyedmers et al., 2008). Since some chaperone proteins were shown to bind to aggregates of misfolded proteins, one could expect that GFP fusions of such chaperones would decorate aggregates formed in response to hyperosmotic shock, thus allowing monitoring of aggregate formation *in vivo*. To do this, the strains from the yeast GFP fusion collection (Huh et al., 2003) containing tagged Hsp104 and Ssa1 chaperones, which bind both to amorphous and amyloid aggregates (Chernoff et al., 1995; Glover and Lindquist, 1998) and are expressed at relatively high levels, were initially used. In order to study protein aggregation in response to hyperosmotic shock, we monitored the localization of these proteins, as well as several other chaperones. We observed that they formed numerous foci, which we termed OSFs (Fig. 1), in response to various types of hyperosmotic shock (KCl and sorbitol in culture medium). Further experiments with Ssa1, unless noted otherwise, were performed using the Ssa1-Dendra2 (Ssa1-DDR) fusion protein, which behaved identically to Ssa1-GFP in terms of OSF formation, but did not form single large and stable inclusions similar to those observed by Kaganovich et al., (2008), which complicated OSF visualization. Also, KCl was used as the hyperosmotic agent in further experiments.

Ssa1-DDR formed OSFs efficiently only under specific cultivation conditions. Initially we observed that cells grown to high density in rich medium with glucose as the sole carbon source (YPD, with D denoting dextrose) formed OSFs efficiently, while cells of logarithmic cultures were not capable of forming OSFs or formed indistinct OSFs (Fig. 1B,D). This suggested that OSF formation by Ssa1 was dependent on the carbon source, since upon reaching a certain density a yeast culture depletes glucose in the medium by converting it to ethanol, at which point the culture experiences the so-called diauxic shift, and starts consuming ethanol generated during glucose fermentation. In agreement with this suggestion, cells producing Ssa1-DDR and Hsp104-GFP on medium with glycerol as the sole carbon source (YP-Gly) formed OSFs efficiently even in logarithmic cultures (Fig. 1B,D). On the other hand, the chaperone Hsp42 formed distinct OSFs not only in cells grown on non-fermentable carbon sources such as glycerol, but also in cells consuming glucose (Fig. 1B). This indicates that OSF formation by specific proteins may require different conditions.

To understand if OSF formation was conserved in at least some other yeast species, we assayed OSF formation by Hsp70 in the distantly-related methylotrophic yeast *Ogataea parapolymorpha*. Microscopic analysis of cells expressing the closest homologue of *Saccharomyces* *cerevisiae* Ssa1 fused to GFP demonstrated that this chimeric protein could also form OSFs when the cells were grown in YP-Gly medium (Fig. 1C,D).

**Fig. 2.**
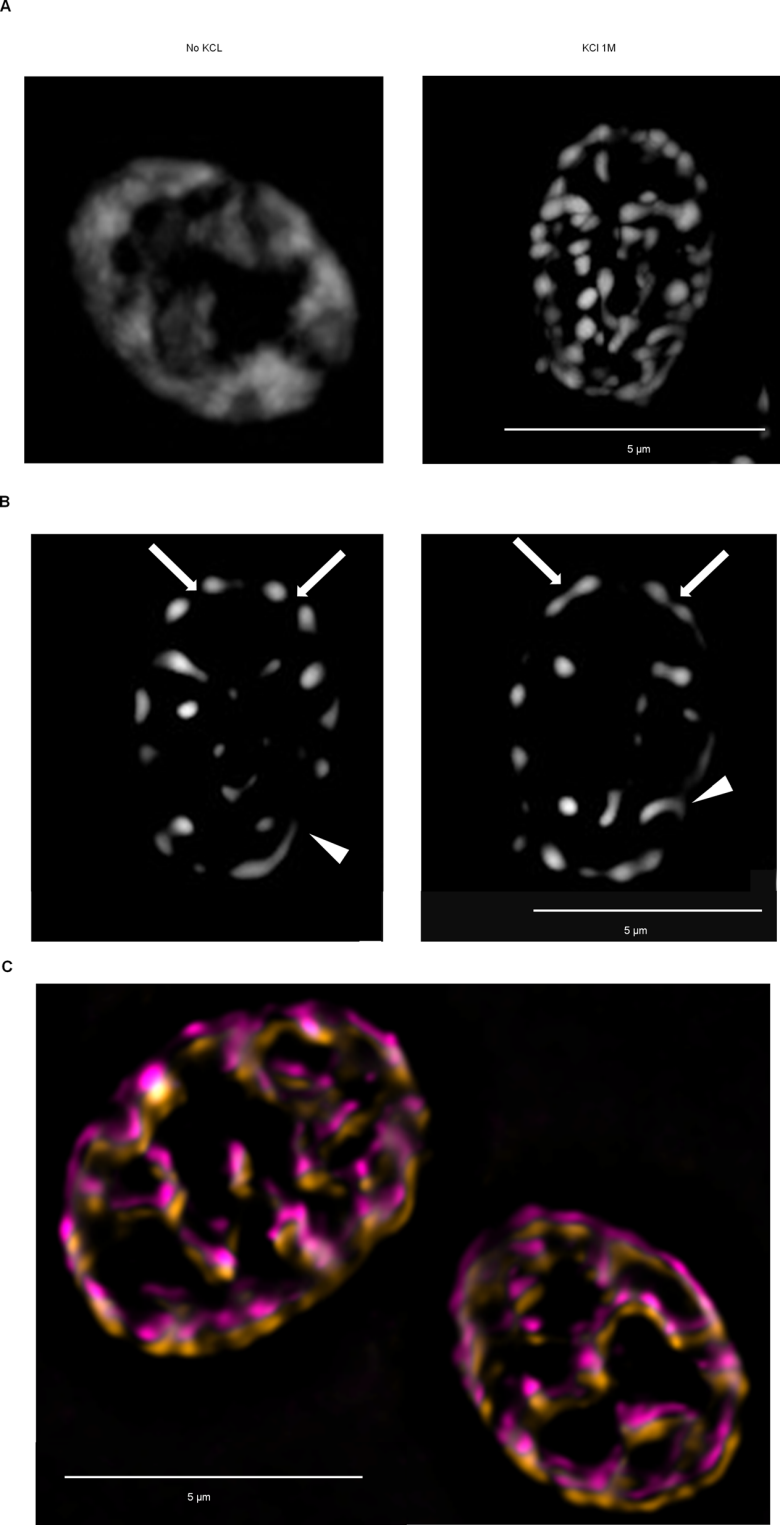
**SIM reveals bridges between OSFs as well as small OSF protuberances.** Cells producing the Ssa1-DDR protein were collected in the logarithmic phase of growth from YP-Gly medium, and either subjected or not subjected to hyperosmotic shock with 1M KCl in the same medium. (A) Maximum intensity projection of a representative shocked and non-shocked cell. (B) Selected optic sections from the same shocked cell shown in A that contain OSFs with protuberances or interconnections between adjacent OSFs. Arrows indicate interconnections, while protuberances are indicated by arrowheads. (C) Two SIM images (average intensity projections) of the same cells taken within a 2-min interval (initial image pseducolored magenta, with orange after 2 min). An offset was introduced in order to facilitate viewing of changes in configuration of features. Scale bars: 5 µm.

**Fig. 3.**
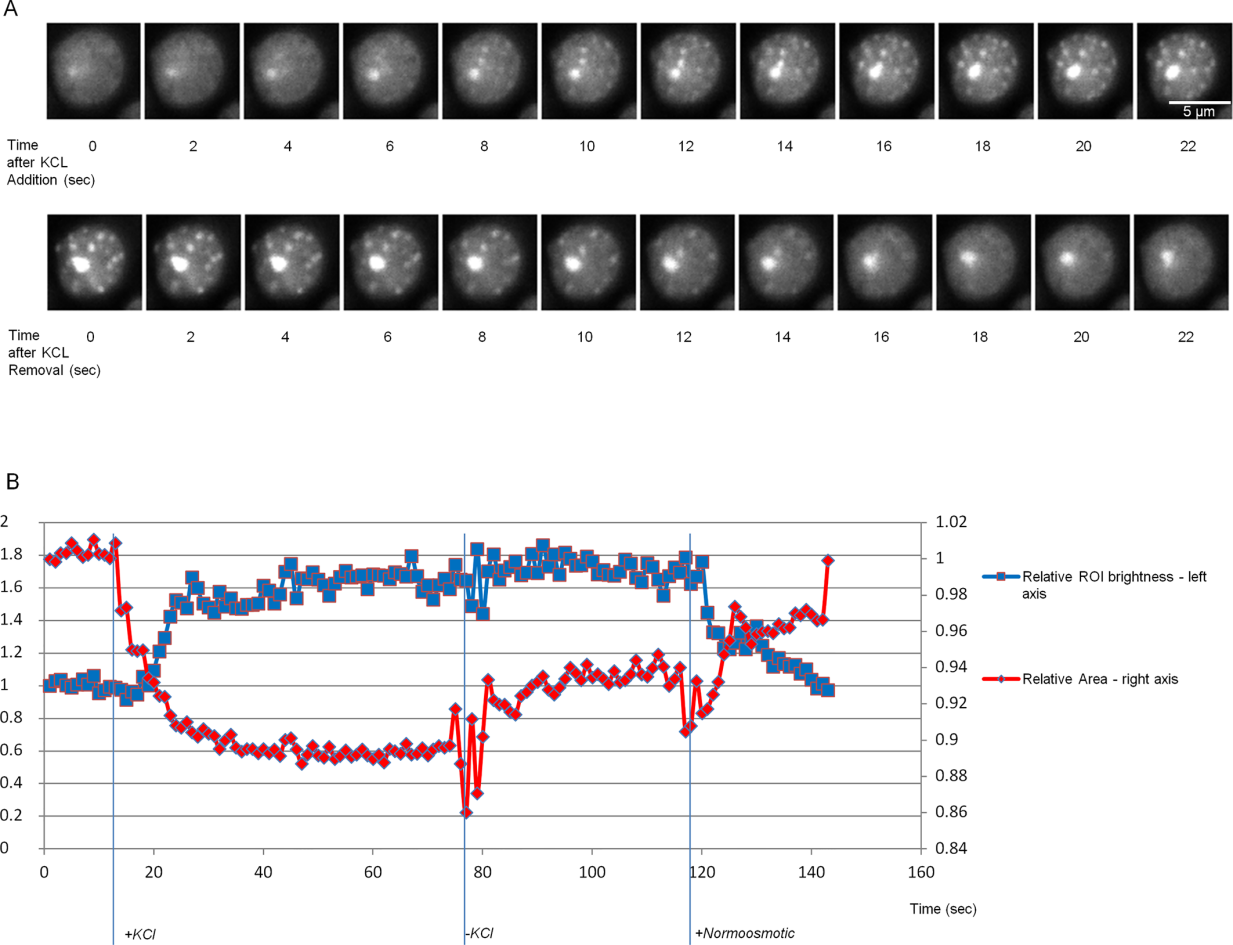
**Dynamics of Ssa1-GFP OSF formation and disappearance.** (A) Time-lapse images of OSF formation and disappearance in response to onset and removal of hyperosmosis, respectively, obtained using cells expressing the Ssa1-GFP protein. Images were obtained using confocal real-time microscopy. Scale bar: 5 µm. (B) Time course of changes in cytoplasmic area and OSF formation/disappearance. Vertical lines depict the time points at which the KCl-induced hyperosmotic shock was administered (+KCl), the time at which medium containing KCl was aspirated (−KCl), as well as the time at which normoosmotic medium was added (+Normoosmotic). The presented graph was obtained from the cell images in A, except with higher temporal resolution. Ten other cells were also used to construct graph with similar results**.** The Ssa1-GFP protein was used instead of Ssa1-DDR to provide a stronger signal without photobleaching. The large aggregate that is constant through both time courses is an IPOD-like inclusion of Ssa1-GFP that has no relation to OSFs. Fluctuation in the graph during KCl removal and slight change of apparent cell area afterwards is due to vibrations of the sample during solution aspiration, which affected microscope focusing.

To observe OSFs at increased resolution, we used structural illumination microscopy (SIM; Gustafsson, 2000; Heintzmann and Cremer, 1999) and subsequent image deconvolution. Interconnections between some of the OSFs became evident, while others exhibited small protuberances which did not contact other foci (Fig. 2). Overall, the OSFs seemed to form a more or less interconnected network in the cell (Fig. 2 and Movie 1). Notably, these networks were relatively stable at minute timescales, since imaging at 2-min intervals showed only minor rearrangement of the OSF network (Fig. 3C).

### OSF formation is rapid and reversible

To determine how fast OSFs form and whether they persist after stress removal, we performed time-lapse microscopy of cells exposed to sequential hyperosmotic and normoosmotic conditions. Cells grown on normosmotic medium were imaged in real time during addition of hyperosmotic agent. Then the hyperosmotic medium was removed and replaced with normosmotic medium. To our surprise, OSF formation was very rapid, i.e. the cells formed OSFs within seconds after exposure to KCl and these OSFs disappeared just as rapidly upon cessation of hyperosmosis (Fig. 3 and Movie 2). This makes it highly unlikely that OSFs represent protein aggregates or stable protein complexes. Next, we used image analysis to estimate the volume of observed cells. We showed that OSFs appear during shrinking after addition of KCl and that, upon shock cessation, the foci disappear concomitantly with cytoplasmic volume restoration (Fig. 3).

### Identification of OSF-forming proteins by screening strains of the GFP-fusion library

Thus, the rapid and reversible formation of OSFs, coupled with their unusual shape, indicates that these structures are likely to be of a soft, probably liquid, nature. Most likely due to these reasons, we failed to isolate OSFs of Ssa1-DDR by centrifugation of lysates of cells (data not shown), even if cell lysates were obtained under increased crowding conditions, which has been shown to facilitate purification of unstable protein complexes (Petrovska et al., 2014). Therefore, in order to identify proteins capable of OSF formation, we used high-throughput microscopy to screen for changes in protein localization in response to hyperosmotic shock in a collection of 4156 strains expressing GFP-fusion proteins (Huh et al., 2003). Cells were grown in YP-Gly medium, washed and then resuspended in 1M KCl or distilled water, transferred to 384-well glass-bottom microscopy plates and analyzed by high-throughput microscopy. This approach restricted adjusting exposure time in each strain, so we chose to use the same exposure time throughout the screen, which could be inappropriate for observation of a number of poorly expressed proteins. Despite these limitations, 10 novel OSF-forming proteins were revealed (Table 1 and Fig. S2). These included proteins of unknown function, metabolic enzymes, a subunit of the translation initiation factor (Clu1) and several chaperones. Notably, the control protein Dendra2 expressed under control of a strong *ADH1* promoter, nor most of the tested GFP fusion proteins including highly expressed ones, such as Pab1-GFP, Pub1-GFP, Tdh1-GFP and two ribosomal proteins did not form OSFs (Fig. S3).

**Table 1.**
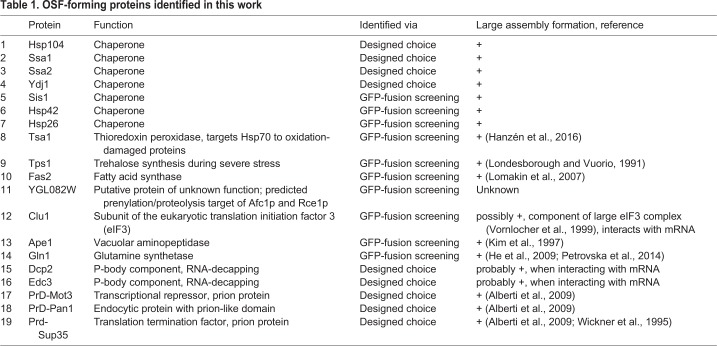
**OSF-forming proteins identified in this work**

**Fig. 4.**
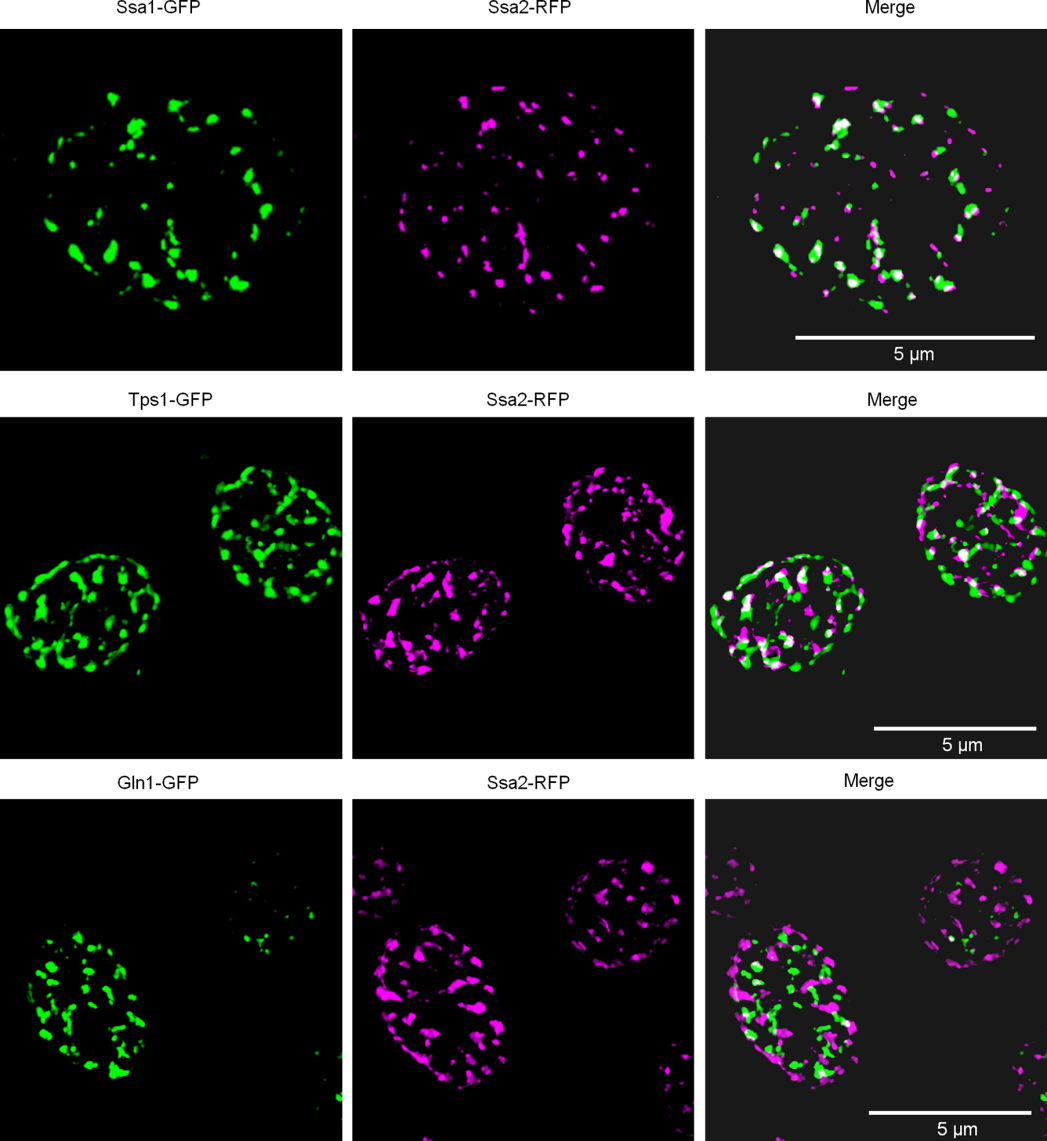
**OSF-forming proteins show only partial or absent colocalization.** Diploid cells expressing pairs of the indicated GFP/tagRFP-tagged proteins were grown to mid-log phase in YP-Gly medium, transferred onto the same medium with 1M KCl and visualized by SIM microscopy. Scale bars: 5 µm.

Analysis of cells expressing pairs of OSF-forming proteins and growing in conditions promoting OSF formation using SIM microscopy showed only partial colocalization in some pairs (Ssa1/Ssa2 and Tps1/Ssa2) or complete lack of it (Gln1/Ssa2) (Fig. 4). This suggests that the OSFs formed by various proteins can be independent structures or differ considerably in the proportion of various proteins. However, partial colocalization suggests that different proteins can also be present in the same foci.

### Identification of additional OSF-forming proteins

**Fig. 5.**
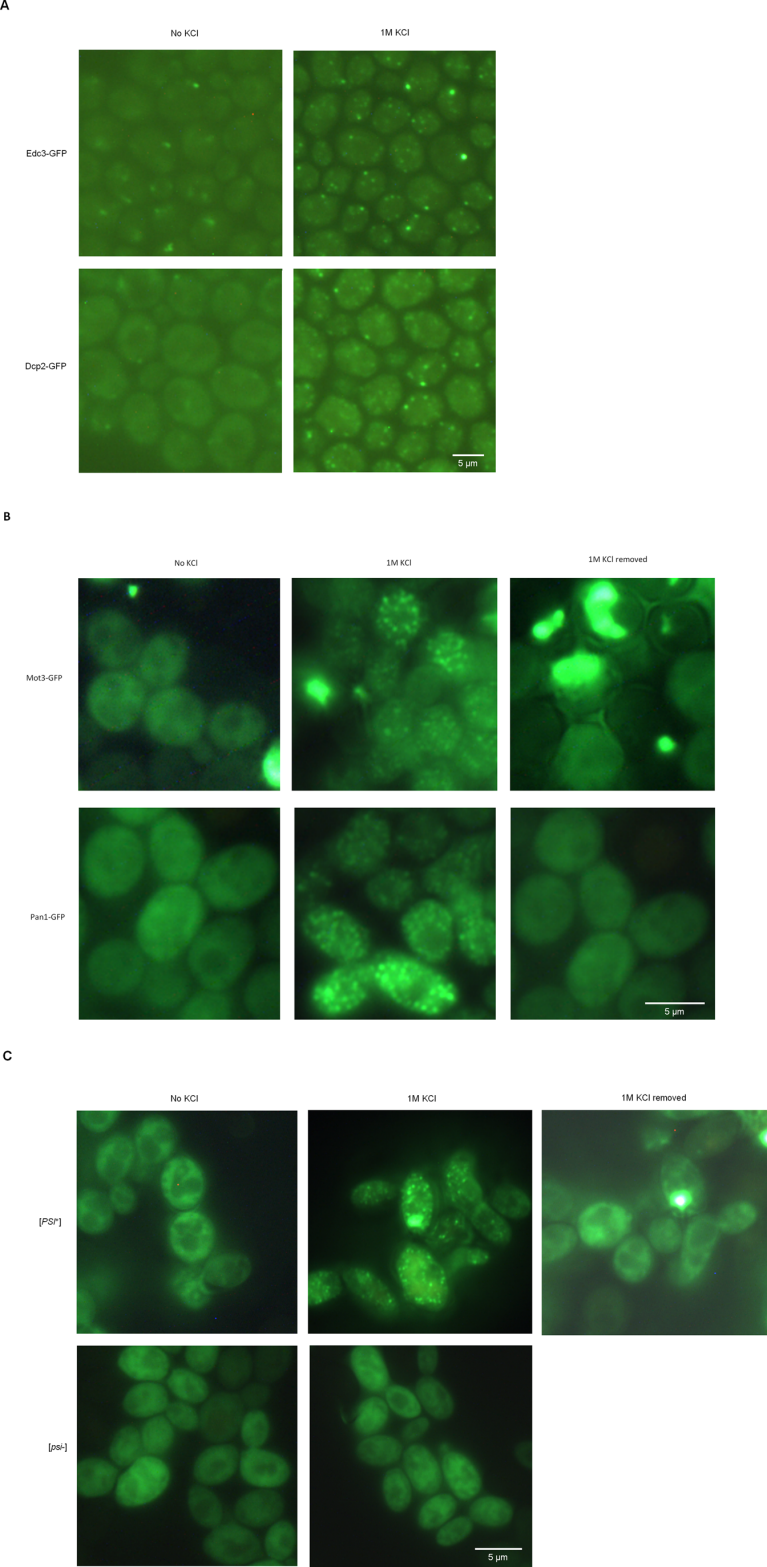
**P-body proteins and proteins with amyloidogenic domains can form OSFs.** (A) Cells producing the indicated GFP-fusion proteins were grown in YP-Gly medium and washed in SC-Gly medium to reduce background medium fluorescence. The cells were subsequently subjected to hyperosmotic shock (1M KCl) in SC-Gly. (B) Cells of the 74D-694 [psi^−^] strain harboring the plasmids expressing PrDMot3-GFP or PrDPan1-GFP were grown in SC-Gal medium to induce GFP-fusion protein production and then transferred onto the same medium supplemented with 1M KCl. (C) Cells of the 74D-694 strain harboring the plasmid expressing PrDSup35-GFP, as well as containing or lacking prion amyloids of Sup35 ([PSI^+^] or [psi^−^], respectively) were grown in SCGly medium and then transferred onto the same medium with 1M KCl. The growth of cells in SCGly was required to observe diffuse fluorescence of the protein in normo-osmotic conditions, since high production of PrdSup35-GFP upon growth of [PSI^+^] cells in SC-Gal resulted in almost complete accumulation of this protein in IPOD-like inclusions. Due to the presence of the ade1-14 mutation, additional adenine was added to the medium to reduce of the amount of autofluorescent red pigment accumulated in cells of the ade1 mutants. Scale bars: 5 µm.

Earlier work has described that upon hyperosmotic shock, some of the stress-granule and processing-body (P-body) proteins can form foci, which are assumed to be P-bodies (Huch and Nissan, 2017; Ramachandran et al., 2011; Teixeira et al., 2005). In agreement with this, we observed that the P-body marker proteins Dcp2 and Edc3 tagged with GFP formed foci (Fig. 5A), though stress-granule markers Pub1 and Pab1 did not (Fig. S2). Even though the P-body proteins were not highly expressed, we still managed to detect OSF-like foci after approximately 30 s of exposure to 1M KCl. Notably, previous work on P-body formation in hyperosmotic conditions did not use such short shock-exposure times. Importantly, the dynamics of OSF formation/disappearance by Dcp2-GFP and Edc3-GFP were so rapid that they were difficult to equate to the much more stable P-bodies that appear in response of glucose deprivation in culture medium (Teixeira et al., 2005), thus indicating that the P-body protein foci that form during hyperosmotic shock may not be genuine P-bodies. More specifically, we suggest that some large, but microscopically unobservable complexes related to mRNA processing may be constantly present in the cell, and hyperosmotic shock causes concentration of these complexes (see Discussion).

Formation of numerous small foci in response to hyperosmosis has been reported for the interacting transcriptional regulators Tup1 and Сyc8 (Han and Emr, 2011; Oeser et al., 2016). Notably, formation of foci for a mutant form of Cyc8 was dependent on its Q/N-rich domain (Oeser et al., 2016), which is required for amyloid formation (Patel et al., 2009). To determine whether other amyloidogenic proteins could form OSFs, we assayed OSF formation by overproduced prion and prion-like domains (PrD) of three such proteins fused to GFP, namely PrDSup35-GFP, PrDMot3-GFP and PrDPan1-GFP. These proteins were capable of forming OSFs similar to those of Ssa1-DDR OSFs in terms of their rapid formation and disassembly (Fig. 5B,C). The rapid disassembly of these OSFs was especially surprising, since it indicated that even though these proteins can form amyloid aggregates upon overproduction (Alberti et al., 2009), the OSFs which they formed were not stable.

### Large complex formation is required for formation of OSFs by some proteins

Notably, PrD-Sup35-GFP was able to form OSFs only in the cells possessing prion determinant [*PSI^+^*], which implies involvement of this protein in prion polymers (Fig. 5C). This suggests that OSFs are formed only by proteins that are members of large complexes, while monomeric proteins do not exhibit such behavior.

Perusal of the data on the identified proteins showed that nearly all of the identified proteins could form large protein assemblies (See Table 1 and references therein and Discussion) in the megadalton range. The OSF-forming proteins that we had identified were especially enriched in chaperone proteins, and we had already observed that growth on a non-fermentable carbon source stimulated OSF formation by Ssa1. Such an environment is known to increase levels of oxidatively damaged proteins (Vasylkovska et al., 2015), so we reasoned that for chaperones, stressful conditions might increase the amount of chaperone bound to damaged proteins and thus increase the share of chaperone involved in the formation of large protein assemblies, stimulating the ability to form OSFs.

**Fig. 6.**
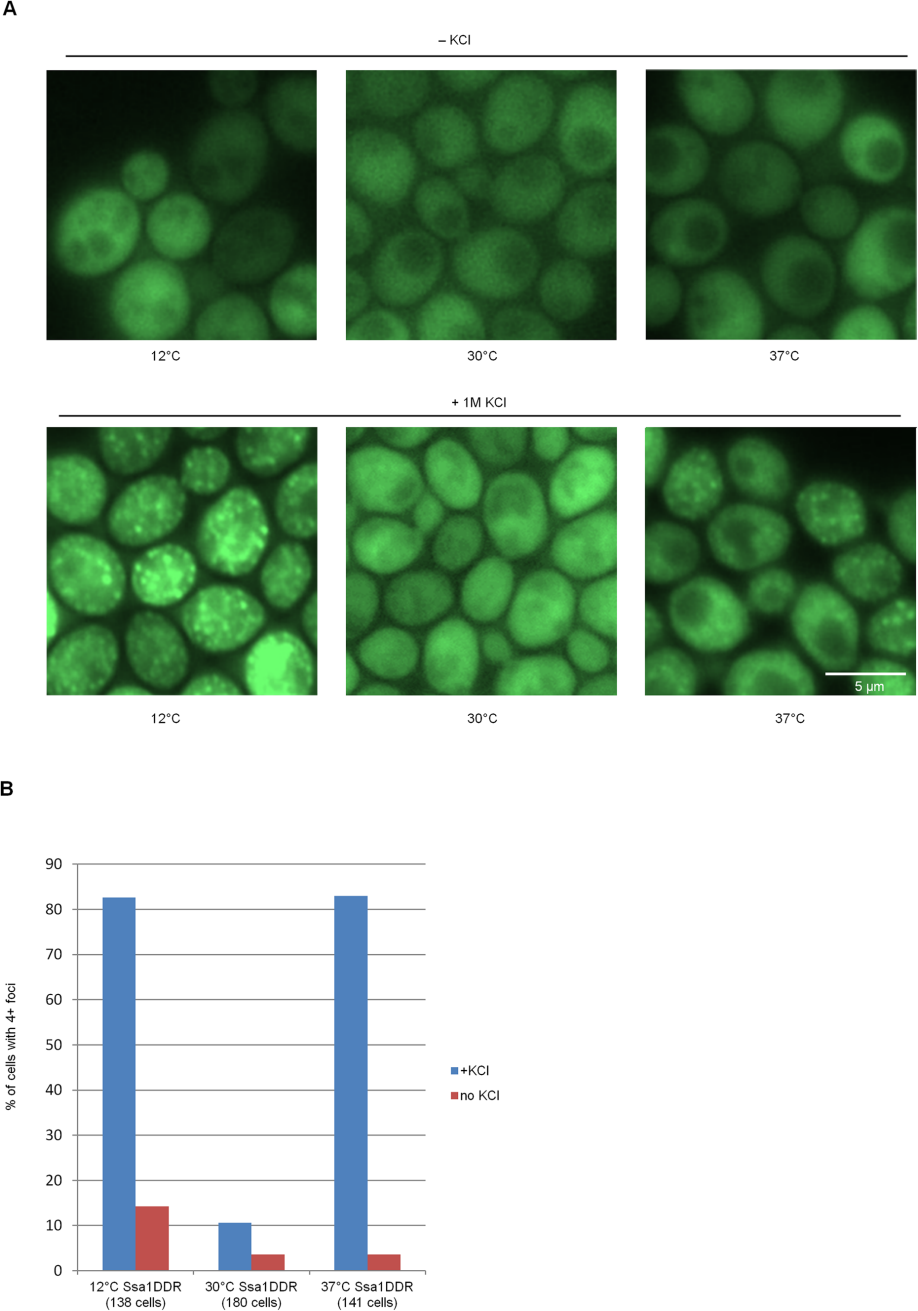
**Conditions of mild stress during growth stimulate formation of OSFs by Ssa1-DDR.** (A) Cells of the BY4741 strain bearing the Ssa1-DDR fusion protein were grown in YPD under the indicated conditions and then subjected to hyperosmotic shock. (B) Analysis of foci numbers in populations of cells used in A, with the y-axis depicting the percentage of cells containing 4+ foci. Exposition times were adjusted to have approximately equal intensity in the various samples. Scale bar: 5 µm.

To test this, we assayed how heat stress (37°C) and cold stress (12°C) affected OSF formation and discovered that these treatments stimulated formation of OSF (Fig. 6), while other treatments such as ER-stress did not (data not shown).

### Screening of the yeast gene knockout/knockdown strain libraries for genes essential for OSF formation

To search for genes required for OSF formation by Ssa1, we used the Synthetic Genetic Array (SGA) technique (Tong and Boone, 2006) to introduce Ssa1-DDR into strains of the commercially available Yeast Deletion (Giaever et al., 2002) and Yeast DAmP collections (Breslow et al., 2008). We then used the resulting array of strains to search for gene deletions or knockdowns which prevent formation of Ssa1-DDR OSFs in hyperosmotic conditions. Approximately 6000 strains were grown on YP-Gly plates, transferred to 1M KCl and then subjected to high-throughput microscopy. After obtaining preliminary results and rechecking the observed phenotypes of strains, we could not identify any genes which reproducibly prevented OSF formation. This might suggest that OSF formation may be a physical phenomenon not controlled by any single specific gene.

## DISCUSSION

In this work, we discovered a novel type of cellular response to hyperosmotic shock – the nearly instant and reversible appearance of foci formed by GFP-labeled proteins and identified a number of proteins that form these OSFs. Intriguingly, the fast dynamics of OSF formation and disassembly, as well as tight correlation of their appearance and disappearance with changes of the cytoplasmic volume, make it unlikely that OSFs represent conventional protein aggregates or stable protein complexes. This conclusion is also supported by the observation that even though Sup35 and Mot3 proteins are capable of forming highly stable amyloid aggregates (Alberti et al., 2009), they form reversible OSFs which are disassembled in a matter of seconds after shock removal.

Of the possible models which could explain our data, the process of liquid–liquid phase separation, which has attracted much interest in the last few years, might be relevant. In frame of this process, OSFs could be liquid droplets suspended in the cytosol, which form rapidly due to any of the following factors that accompany hyperosmotic shock: increased protein or ion concentrations, as well as increased crowding, which have all been demonstrated to affect phase separation (Delarue et al., 2018; Kaur et al., 2019; Lemetti et al., 2019). Notably, a recent preprint on BioRXiv reports foci similar to OSFs formed by the YAP protein in response to hyperosmosis in mammalian cells (Cai et al., 2018 preprint). The authors base their conclusions of the phase-separated nature of YAP foci on the observations that the foci are round and that they are able to fuse with each other. In our case, the foci were mostly immobile and often exhibited stable elongated shapes and interconnecting bridges between foci (Fig. 2). Notably, this does not necessarily mean that OSFs are not phase-separated droplets, however, if they are, they are being deformed by their surroundings and are trapped between other entities.

We also envisioned an alternative and possibly somewhat simpler explanation. This was based on the fact that increased environmental osmolarity causes water efflux from the cytoplasm and a concomitant decrease in cellular and cytoplasmic volume. Since OSF proteins are primarily cytosolic, but most of the cytosolic proteins we observed did not form OSFs, we posit that the cytosol is structured in a way that its components are differentially affected by hyperosmotic shock. Specifically, it could be that some areas of the cytosol easily lose water (we term these areas ‘liquid’) and some do so less readily (‘solid’), possibly due to structural rigidity. Then such areas could have different effects on cytosolic proteins, if some proteins could easily travel between the liquid and solid areas, while other could not. For brevity, we will call this the ‘beads in liquid’ model.

**Fig. 7.**
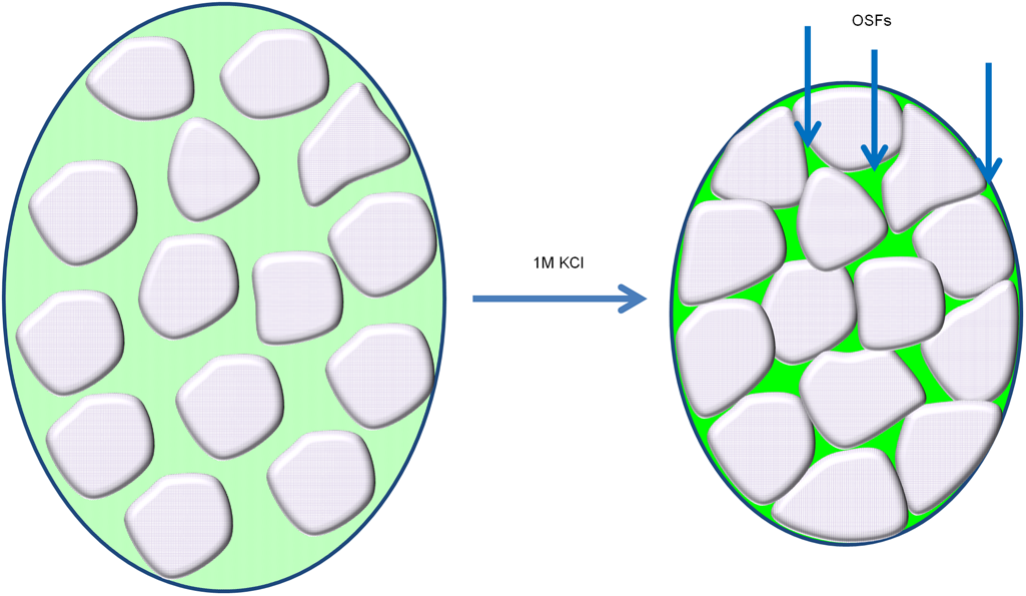
**Schematic representation of the proposed mechanism of OSF emergence during hyperosmotic shock.** In response to hyperosmosis, the cell loses water, concentrating the liquid part of the cytoplasm (green). Putative solid components bunch together, forming concentrated pockets of liquid (more vivid green), that we propose to correspond to OSFs. Nature of solid components may vary, including membrane organelles in part, but also unknown cytosolic structures.

During hyperosmosis water should preferentially escape from ‘liquid’ parts of the cell but not from its more ‘solid’ areas. Thus, ‘liquid’ areas of the cytoplasm should decrease in volume, forcing ‘solid’ areas, as well as some organelles, to come into closer contact, thus creating pockets of liquid (Fig. 7). While all of the cytosolic components increase in concentration due to reduction of cellular volume, the increase in the concentration of components trapped in the ‘liquid’ network will be much more drastic, since they are unable to access the considerable volume represented by the ‘solid’ areas. So, with GFP labeling, this would make these pockets of concentrated liquid small and bright (microscopically observable as OSFs). This idea is compatible with liquid–liquid phase separation, i.e. the phase-separated droplets could be forming in the concentrated pockets of liquid, however, a simpler explanation is that the OSFs may in fact be the liquid pockets themselves. It is currently unclear what the gel-like ‘solid’ regions might constitute, however the work of (Delarue et al., 2018), which demonstrates the role of ribosomes as a crowding factor in the cytosol, and our results concerning the inability of ribosome proteins to exhibit OSF behavior (Fig. S1) might suggest that ribosomes, especially in their polyribosome form, might be a candidate for these entities.

Notably, fusion of foci, which is a commonly accepted indicator of liquid–liquid phase-separated nature of foci, is also likely in the suggested model, because shifting of ‘solid’ entities during or after water efflux should result in an effect that could look like fusion, even though it actually may be liquid network rearrangement.

Rapid formation and disappearance are also inherent features of this model, since no processes other than change of cytoplasmic volume are required. We must note however, that LLPS can also proceed very quickly under certain circumstances (Bracha et al., 2018). That said, we do not know of any data concerning the maximal rates of disappearance of phase-separated droplets. The morphology of OSFs revealed by high-resolution microscopy is also consistent with the ‘beads in liquid’ model, since pockets of liquid formed due to the bunching together of solid compartments would be expected to look like the interconnected OSF network observed by SIM (Fig. 2 and Movie 1). Finally, screening for gene deletions and knockdowns interfering with Ssa1 OSF appearance revealed no single deletions that significantly impacted the OSF-forming behavior of Ssa1-DDR, which is consistent with the idea that OSFs are a consequence of cytoplasmic volume reduction, which is unlikely to be controlled by the action of single genes.

Our ‘beads in liquid’ model implies that OSF formation should be characteristic of proteins that are trapped in the liquid phase, i.e. for proteins which cannot travel freely between the liquid and ‘solid’ cytoplasmic compartments. Since various studies (Delarue et al., 2018; Guo et al., 2014; Luby-Phelps, 1999) have demonstrated that large complexes exhibit anomalous subdiffusion in cells, and our data suggest that aggregated or aggregate-interacting proteins (amyloids and chaperones) or those forming multimeric complexes (see Table 1) form OSFs, it seems likely that a size threshold is one of the parameters that can control trapping in the liquid phase.

According to our model, concentrated pockets of liquid should form under any of the conditions tested, as they are a fundamental physical response of the cytosol to hyperosmotic shock, however the work of Delarue et al. (2018) indicates that ribosome concentrations could also play some role in the process, since the volume of the liquid phase would depend on the amount of ribosome crowding. However, observation of specific proteins in OSFs would also depend on whether they are involved in large complexes that exhibit the ability to be trapped in the liquid phase under given conditions, as well as on the ratio between high and low molecular weight complexes of the protein. Indeed, we observed that conditions preventing OSF participation for Ssa1 (growth on YPD at 30°C) still allowed OSF participation for Hsp42 (Fig. 1B).

The general view on the structure of the cytosol ranges from a simple solution to a crowded liquid, to a gel, and recent data also report on the ability of the cytoplasm as a whole to transition between liquid (characterized by rapid diffusion) and glass-like states (Joyner et al., 2016; Munder et al., 2016; Parry et al., 2014). Our model is in line with observations indicating that the cytoplasm is not homogeneous and contains areas with different diffusion properties (Feric et al., 2016; Luby-Phelps, 1999). Indeed, recent work, which used fluorescent correlation spectroscopy to construct diffusion maps of several cytoplasmic proteins, showed that the cytoplasm is not uniform in terms of protein diffusion rates (Ranjit et al., 2014). The three above reports were performed using cells of higher eukaryotes, and in most cases the observed effects were attributed to structures formed by the actin cytoskeleton (Feric et al., 2016; Guo et al., 2014). However, OSF formation was not affected by disassembling the actin cytoskeleton with latruncullin A or by benomyl, a tubulin depolymerizing agent (data not shown), and subdiffusive properties of large protein complexes observed by (Delarue et al., 2018) were mostly due to ribosome concentrations and not cytoskeletal effects. Thus it is possible that various types of cytoplasmic structuring can exist.

Further studies are necessary to obtain solid proof for the suggested ‘beads in liquid’ model of cytosolic architecture, determine its relevance for processes which take place in the cytoplasm, as well as identify the nature of the putative solid compartments. Importantly, our discovery provides a model in which all of these directions can be explored.

## MATERIALS AND METHODS

### Yeast strains and cultivation conditions and plasmid construction

Most of the experiments in this work used cells derived from the BY4741 strain (MATa *his3-1 leu2-0 met15-0 ura3-0*), except for the experiments concerning OSFs formed by amyloidogenic proteins, which were performed in 74D-694 (MATa *ade1-14, trp1-289, his3Δ-200, ura3-52, leu2-3,112*) and deletion screening, which was performed on hybrids between BY4741 strains from the Yeast Deletion (Giaever et al., 2002) and Yeast DAmP collections (Breslow et al., 2008) with a SGA query strain Y5563 (MATα *can1*Δ::MFA1pr-HIS3 *lyp1*Δ *ura3*Δ*0 leu2*Δ*0 his3*Δ*1 met15*Δ*0*) (Tong and Boone, 2006). The media used were YPD (yeast extract 1%, peptone 2%, glucose 2%) and YP-Gly (yeast extract 1%, peptone 2%, 2.5% glycerol) and SCD (yeast nitrogen base, 0.17 g/l; ammonium sulfate, 5 g/l; glucose, 2%; casamino acids, 0.5%; tryptophan, 75 mg/l; uracil, 75 mg/l; adenine, 19 mg/l) and SC-Gly (same, except with 2.5% glycerol instead of glucose). When necessary, solid medium was prepared by including 2% agar. Unless noted otherwise, the cells were grown to mid-log phase at a temperature of 30°C.

The *O. parapolymorpha* strain DL1-L (*leu2*), derived from DL-1 (ATCC 26012) was modified to produce Hsp70 tagged with tagGFP. We chose the closest homologue of *S. cerevisiae* Ssa1 (NCBI Ref XP_013937290). A codon-optimized tagGFP coding sequence (ordered from Biomatic, Canada) possessing SalI prior to the the tagGFP ORF was cloned between Asp718 and BglII sites of pAM773 (Agaphonov, 2017). Then, inversed recombination arms directing plasmid integration into the *O. parapolymorpha SSA1* locus were inserted between SalI and EcoRV sites of the resulting plasmid. The fragment with the inversed recombination arms was obtained by PCR using primers DL_SSA_U2 and DL_SSA_L_Xho (see Table S1 for all primer sequences) and SalI-digested and self-ligated *O. parapolymorpha* genomic DNA. The PCR product was cleaved at one side with XhoI to allow ligation with SalI-generated cohesive end of the vector. Prior to yeast transformation the obtained plasmid pAM783 was digested with SalI to obtain the cassette for replacement of the wild-type SSA for SSA-tagGFP fusion according to scheme described previously (Agaphonov et al., 2009) (all of the plasmids used in this work are listed and described in Table S2).

Strains producing tagRFP-fusions were constructed from appropriate GFP-tagged strains by using a universal plasmid that replaces the GFP::*HIS3* cassette from the yeast GFP collection with a tagRFP::*URA3* cassette. The plasmid was constructed by modifying the plasmid pFA6a–GFP(S65T)–His3MX, which contains the *Schizosaccharomyces pombe HIS5* gene, capable of complementing the *S. cerevisiae his3* mutation (Longtine et al., 1998). The SmaI-BsrGI fragment was replaced with the g-block tagRFP_Sc (IDT, USA) possessing a codon-optimized tagRFP sequence. Then the NcoI-ScaI fragment bearing the *HIS5 S. pombe* ORF in the resulting plasmid was replaced with PciI-DraI fragment of the *O. polymorpha URA3* locus. The resulting plasmid, designated as pAM781D, was digested with SalI-ClaI to obtain cassette bearing tagRFP with *URA3* selectable marker flanked with sequences homologous to flanking regions of the GFP(S65T)–*His3MX* encoding module.

Strains for colocalization studies were obtained by switching the mating type of the obtained tagRFP-fusion strain with pGal-HO, a plasmid encoding the HO-endonuclease gene under control of an inducible *GAL1* promoter, verifying mating-type change, and crossing it with appropriate GFP-fusion strains. Diploids were selected on SC–His,-Ura medium.

The strains producing Ssa1-DDR were created by PCR-based endogenous tagging of BY4741 using PCR products obtained using primers Ssa1ET-D and Ssa1ET-R with the plasmid 127-DDR-hph used as a templates. The plasmid was a kind gift from Dr Daniel Kaganovich (University of Jerusalem) and papers describing them are currently in preparation. The SGA query strain producing Ssa1-DDR was constructed using an identical procedure.

Plasmids encoding amyloidogenic domains tagged with GFP were constructed using the plasmid pYES2-Sup35NMH-GFP, which was created by inserting the BglII-XbaI fragment encoding Sup35(1-239)-HHHHHHPVAT-eGFP into the BamHI and XbaI sites of pYES2 (Invitrogen). Genomic DNA fragments encoding first 297 amino acid residues of Mot3 and first 223 residues of Pan1 were amplified with primers Mot3-DF, Mot3-Rf, Pan1-Df and Pan1-Rf (see Table S1), and inserted into PvuII/BalI treated pYES2-Sup35NMH-GFP using quick-fusion cloning kit (Bimake) to replace fragment encoding Sup35 residues 1–154 (see Fig. S3 for maps of plasmids).

### Sample preparation for microscopy

To visualize OSFs, cells were spotted onto a 2% agar pad based on SC medium containing 1M KCL (Alexandrov and Dergalev, 2019). Identical pads without KCl were used as a control. Time-lapse videos and SIM images were acquired using glass-bottom plates treated with concanavalin A in order to prevent cell movement.

### Confocal microscopy for real-time imaging

Images were acquired using a dual point-scanning Nikon A1R-si microscope equipped with a PInano Piezo stage (MCL), using a 60× PlanApo VC oil objective numerical aperture (NA)=1.40.

### High-throughput microscopy

Cells were grown in deep-well 96-well plates in YP-Gly medium, washed with YP-Gly and moved to 384 glass-bottom plates (CellVis, P384-1.5H-N, USA). Each sample was placed into two adjacent wells and after loading, KCl was added up to a final concentration of 1M to one of the wells in a pair.

Imaging was performed using an ImageXpress Micro XL (Molecular Devices, USA) high-throughput microscope equipped with a 100× LWD NA=0.9. Three images were collected for each well. Images were viewed manually, and all strains exhibiting shock-induced protein relocalization were reassessed using a manual Zeiss AxioSkop 40 microscope, 100× oil immersion objective, NA=1.2.

### Structural illumination microscopy

Live imaging was performed on an N-SIM super-resolution system (Nikon, Japan) equipped with 100× Plan Apo TIRF lens (NA=1.49) and iXon 897 EM-CCD camera (effective pixel size 63 nm) (Andor, Ireland) in 3D-SIM mode (excitation laser line 488 nm, 120 nm Z-steps) under control of NIS-Elements 4.6 software. Raw image stacks (3 grating angles×5 phase shifts) were analyzed for image quality with the SIMcheck module of ImageJ software and processed using SIM module of NIS-Elements using parameters selected on the basis of Fourier transform analysis. Reconstructed stacks were further deconvolved using the Richardson-Lucy algorithm built into NIS-Elements.

Images for colocalization were processed using FIJI (Schindelin et al., 2012; Schneider et al., 2012), including the Enhance Contrast feature to correct for bleaching and the Maximum Instensity projection feature for presentation in the paper.

### Cytoplasmic shrinkage measurements

Cytoplasmic shrinkage analyses used images obtained from a time-lapse of OSF formation and dissolution obtained on a confocal microscope (see Confocal microscopy for real-time imaging, above). Using ImageJ software, single cells from the time-lapse series were transformed into shapes using the Threshold function and then the areas of these shapes were calculated using the magic wand feature. OSF formation was quantified and graphed by selecting a region of interest which contained a single OSF and the relative signal intensity in this region of interest at different time points was plotted onto a graph together with the change in cell area.

### Deletion screening

The query strain for the Synthetic Genetic Array producing Ssa1-DDR was used to obtain an SGA-array (Tong and Boone, 2006) using the yeast deletion collection and the Yeast DaMP (Breslow et al., 2008) collection in 384-well format. Briefly, this involved mating, sporulation and subsequent selection steps for obtaining haploid progeny of a specified mating type and containing both the gene deletions from the collection and the marker of interest (SSA1-DDR::HygR). The arrayed strains were then grown overnight on YPD plates and then inoculated into 384-well microscopy plates containing SC medium with glucose as a sole carbon source and 1M KCl. The plates were then imaged on a high-throughput microscope, as detailed in the High-throughput microscopy section, above. All the images were then inspected manually in order to detect candidates. Strains which were judged to be defective in OSF formation were reassessed using a manual Zeiss AxioSkop 40 microscope, 100× oil immersion objective, NA=1.3.

## Supplementary Material

Supplementary information
