# Supplementary material for: Analysis of novel hyperosmotic shock response suggests ‘beads in liquid’ cytosol structure
Source: Biol Open. 2019 Jul 8;8(7):bio044529. doi: 10.1242/bio.044529 (PMC6679407; doi:10.1242/bio.044529)
Supplement: Supplementary information [file biolopen-8-044529-s1.pdf]

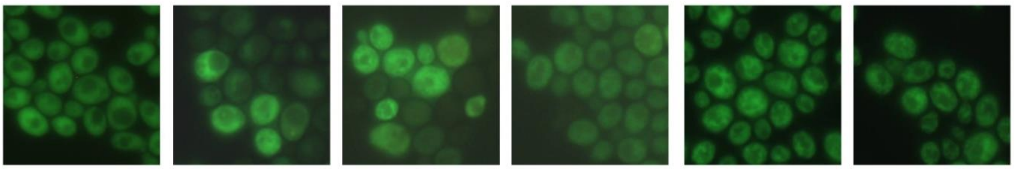

Figure S1. Examples of cytoplasmic proteins that do not form OSFs. – *Cells producing the indicated GFP fusion proteins were grown on YP-Gly medium to log phase and transferred onto the same medium with 1M KCl.*

Biology Open • Supplementary information

Fas2-GFP

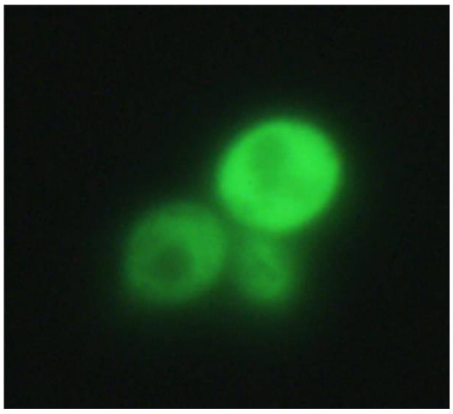

No KCl

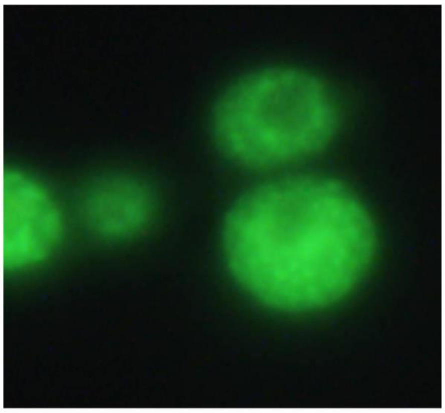

1M KCl

Hsp104-GFP

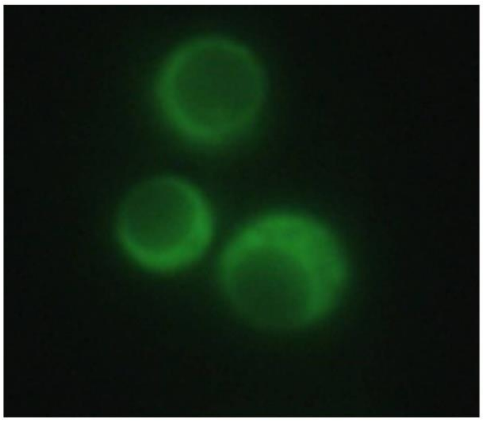

No KCl

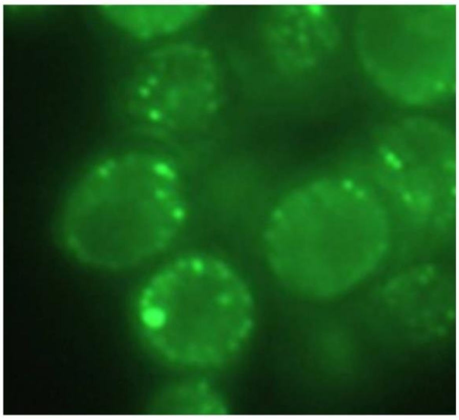

1M KCl

Hsp42-GFP

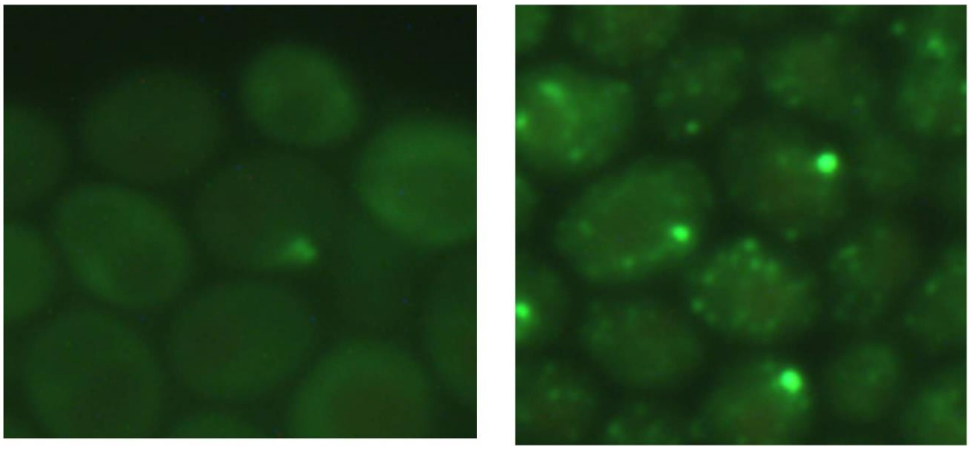

No KCl 1M KCl

Hsp26-GFP

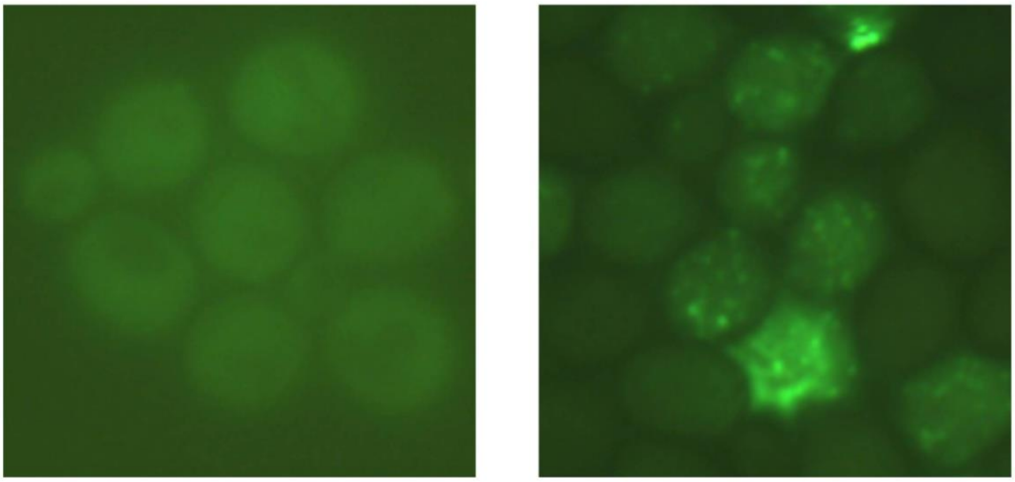

No KCl 1M KCl

Biology Open • Supplementary information

Ssa1-GFP

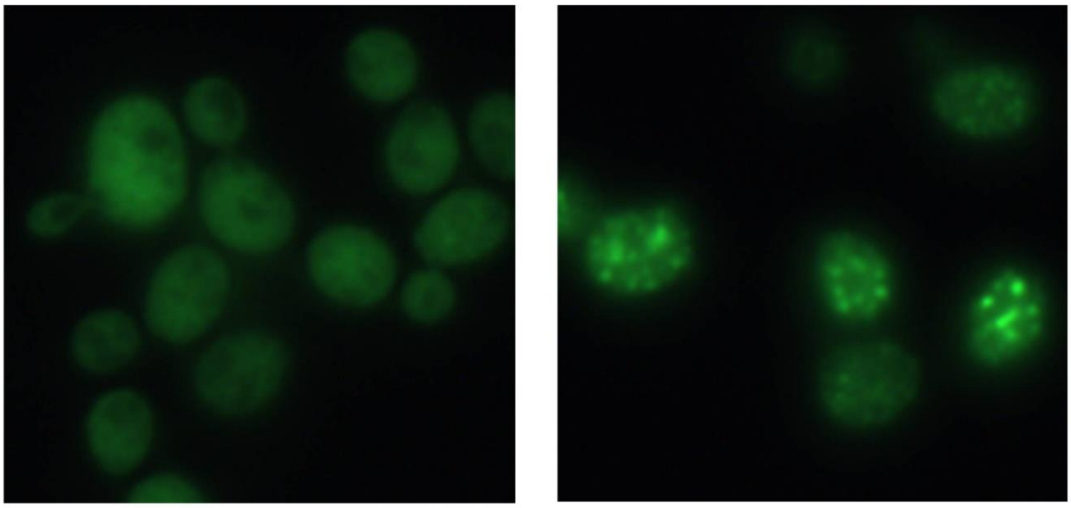

No KCl 1M KCl

Ssa2-GFP

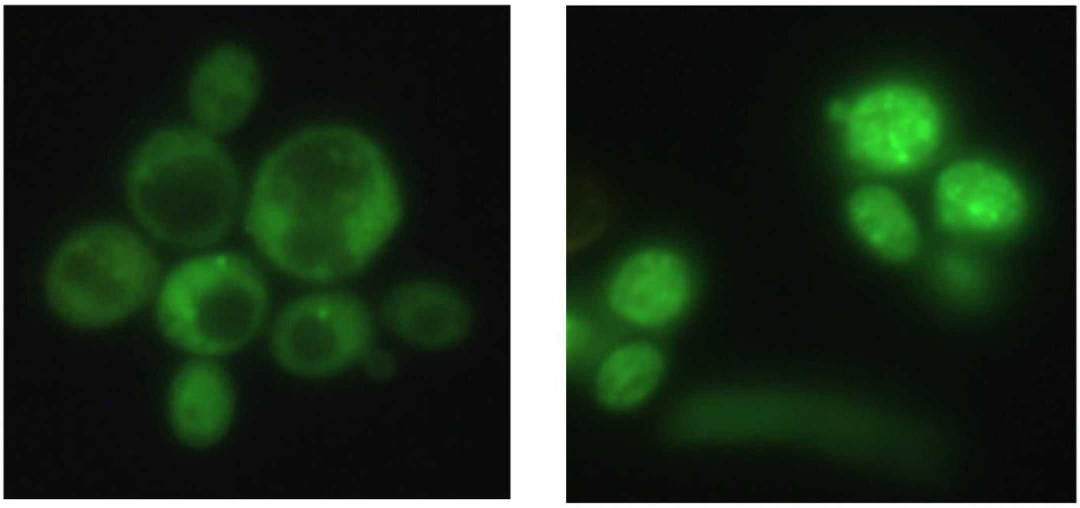

No KCl 1M KCl

Biology Open • Supplementary information

Ydj1-GFP

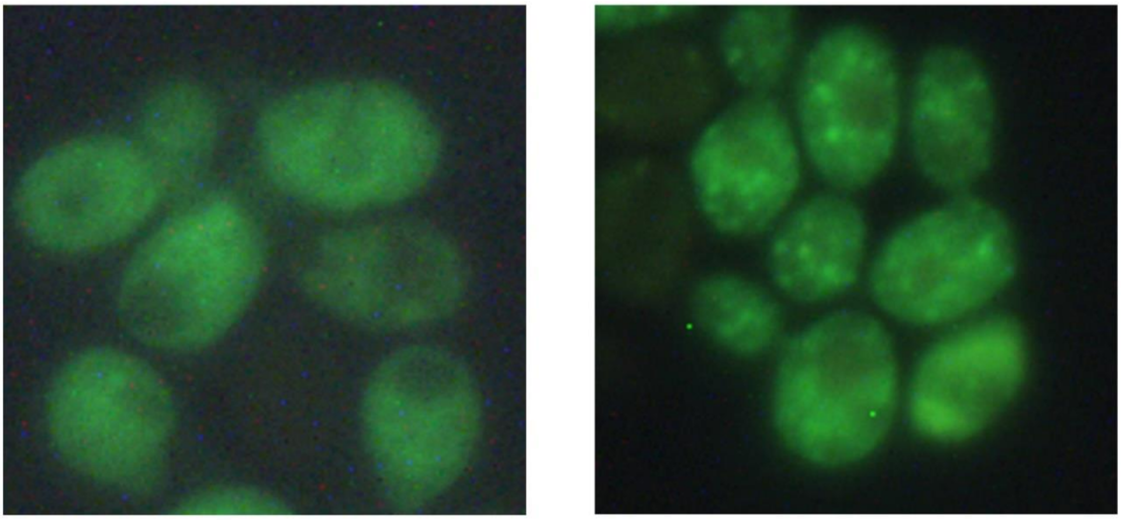

No KCl 1M KCl

Sis1-GFP

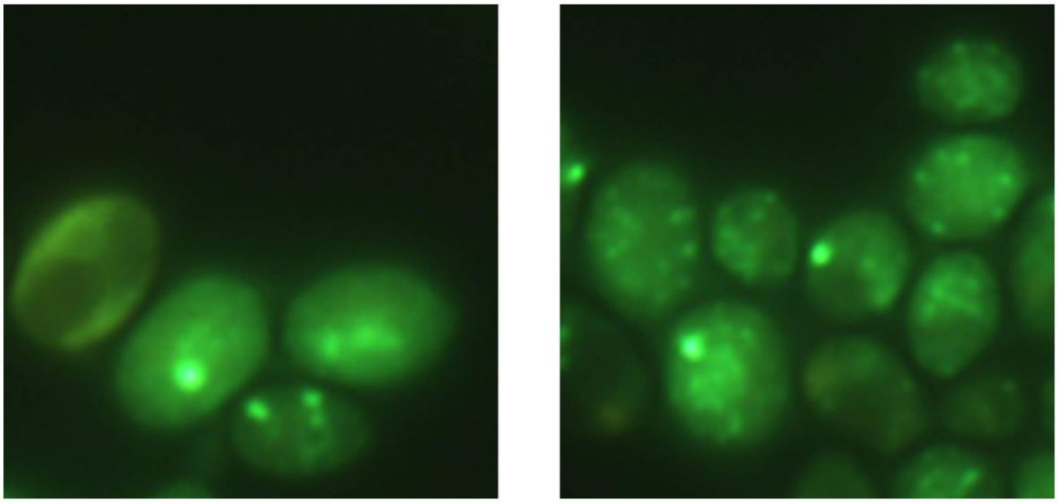

No KCl 1M KCl

Biology Open • Supplementary information

Tsa1-GFP

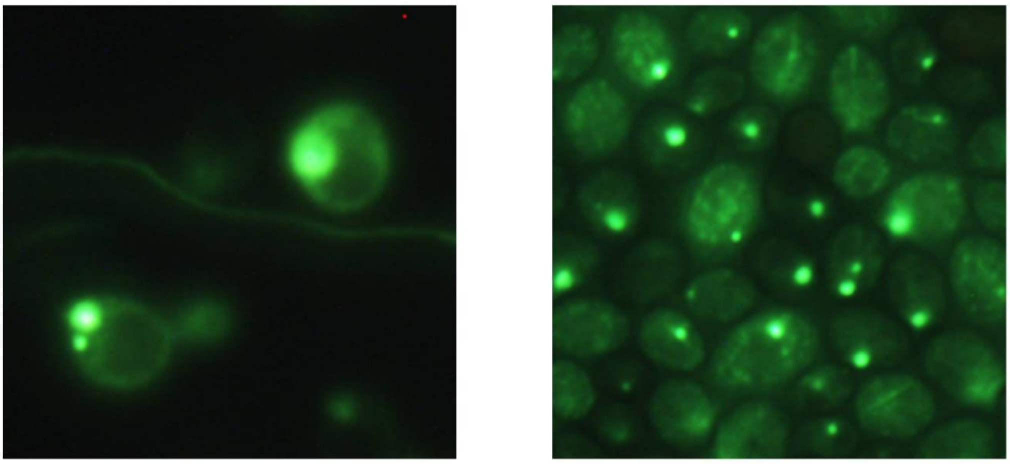

No KCl 1M KCl

Tps1-GFP

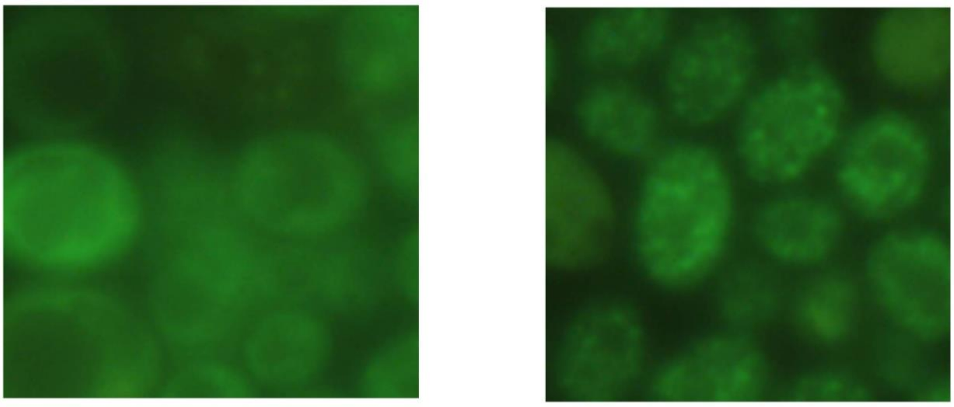

No KCl 1M KCl

Biology Open • Supplementary information

Gln1-GFP

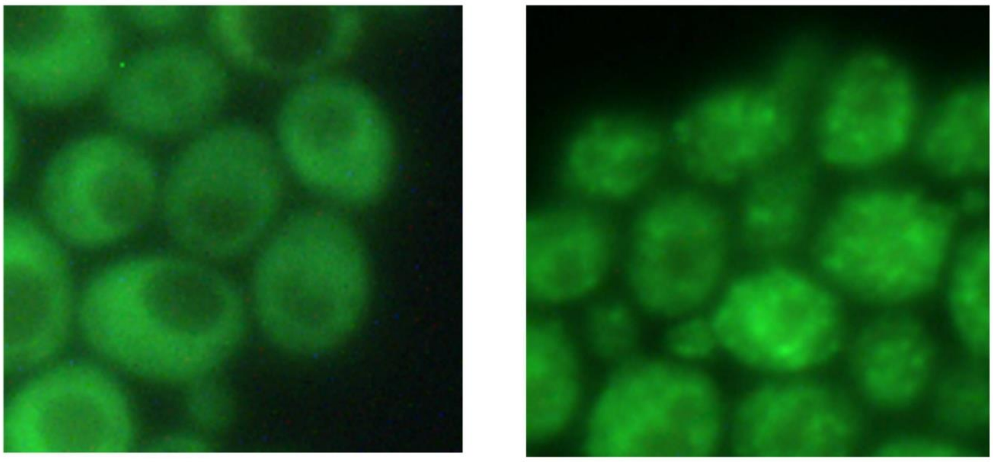

No KCl 1M KCl

YGL082W-GFP

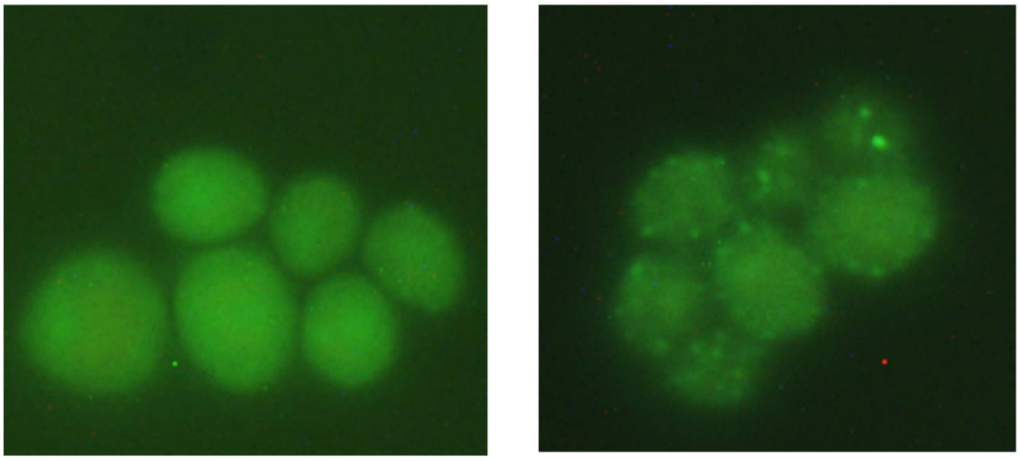

No KCl 1M KCl

Biology Open • Supplementary information

Clu1-GFP

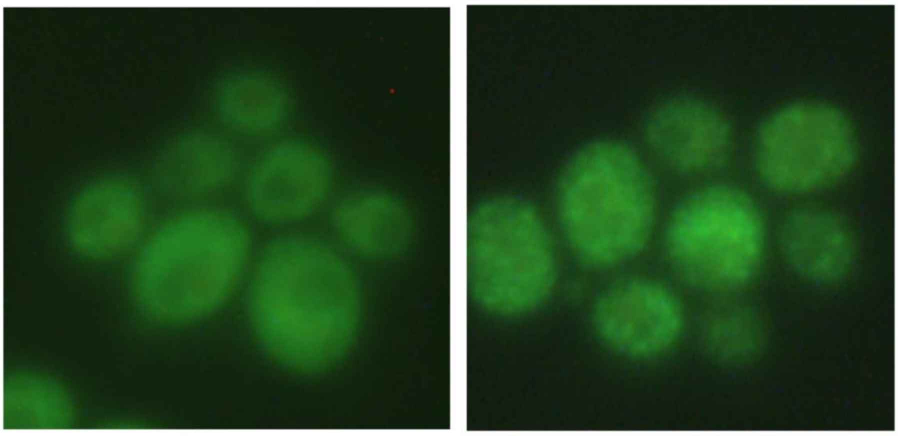

No KCl 1M KCl

Ape1-GFP

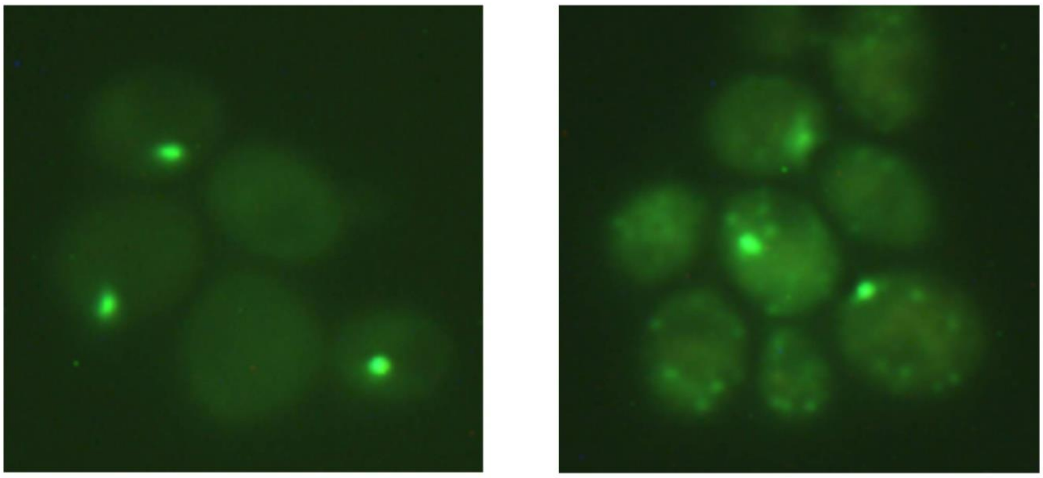

No KCl 1M KCl

Figure S2. Images of strains producing GFP-fusions of various proteins found to form OSFs, without and with hyperosmotic shock (1M KCl).

pAM781D
Map -

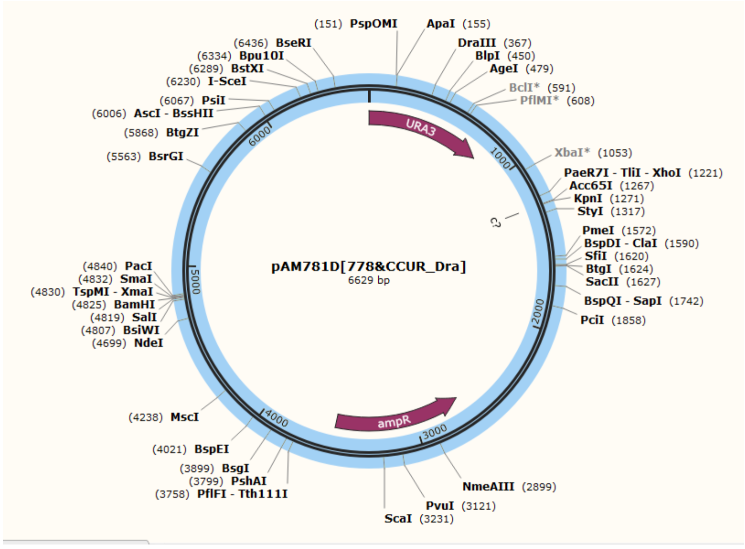

TagGFPopt_BlscSKplusRI_RV
Map

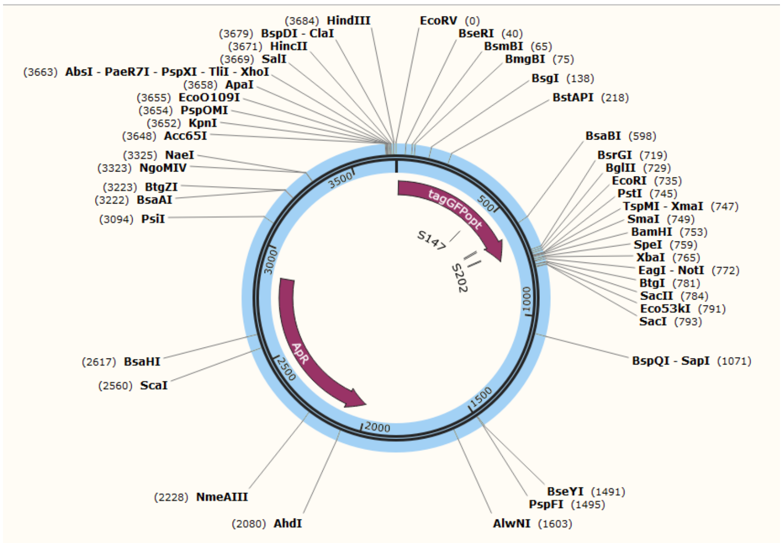

Biology Open • Supplementary information

127-ddr-hph
Map

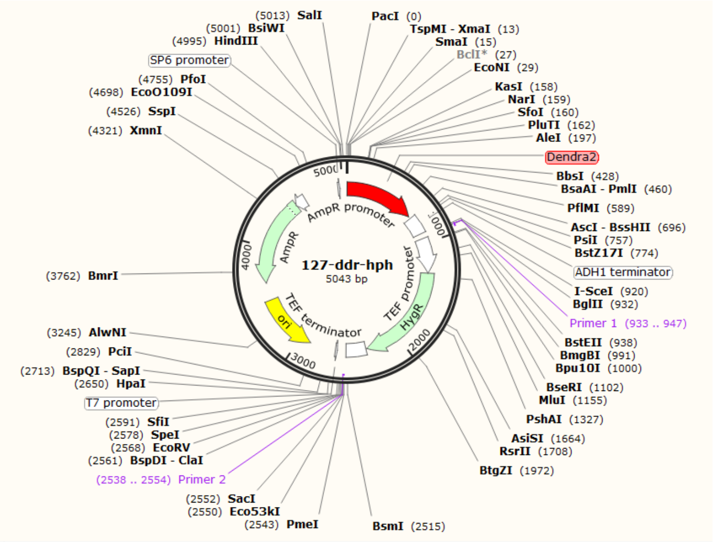

Fig. S3. Plasmid maps.

Biology Open • Supplementary information

Table S1. Primers used in this work

DL_SSA_U2	GGTTGATTAGATATAGAGTAAGCT
DL_SSA_L_Xho	TTCTCGAGATCAACCTCCTCAACAGTTG
Ssa1ET-D	AGCTCCAGAGGCTGAAGGTCCAACCGTTGAAGAAGTTGATGG TGACGGTGCTGGTTTA
Ssa1ET-R	AAAGACATTTTCGTTATTATCAATTGCCGCACCAATTGGCTCG ATGAATTCGAGCTCG
Mot3-Df	ATCGGACTACTAGCAG CAATGAATGCGGACCATC
Mot3-Rf	TTCTTGGTAGCATTGG GCAGAGGTTGTGCCGGGT
Pan1-Df	ATCGGACTACTAGCAG GGCATGGGTTACCAGC
Pan1-Rf	TTCTTGGTAGCATTGG GAGCTTGGTTTTGCGG

Biology Open • Supplementary information

Table S2. Plasmids used in this work.

pAM783	Integrative plasmid for endogenous tagging of O. parapolymorpha Hsp70 with tagGFP	This work
pAM781D	Intergative plasmid for replacement of GFP:: HIS3 module in the GFP Fusion collection with tagRFP::URA3	This work
127-DDR-hph	Template plasmid for endogenous tagging of proteins with DDR2 (HygR selection marker)	Gift from D. Kaganovich
pGal-HO	Autonomous plasmid for mating-type switching, *LEU2* and *URA3* auxotrophic markers, galactose-inducible promoter	unknown source
pYES2-Sup35NMH-GFP	Autonomous multicopy plasmid for expression of the prion domain of Sup35 tagged with GFP, galactose-inducible promoter	This work
pYES2-PrDMot3-GFP	Autonomous multicopy plasmid for expression of the prion domain of Mot3 tagged with GFP, galactose-inducible promoter	This work
pYES2-PrDPan1-GFP	Autonomous multicopy plasmid for expression of the amyloidogenic domain of Pan1 tagged with GFP, galactose-inducible promoter	This work

Biology Open • Supplementary information

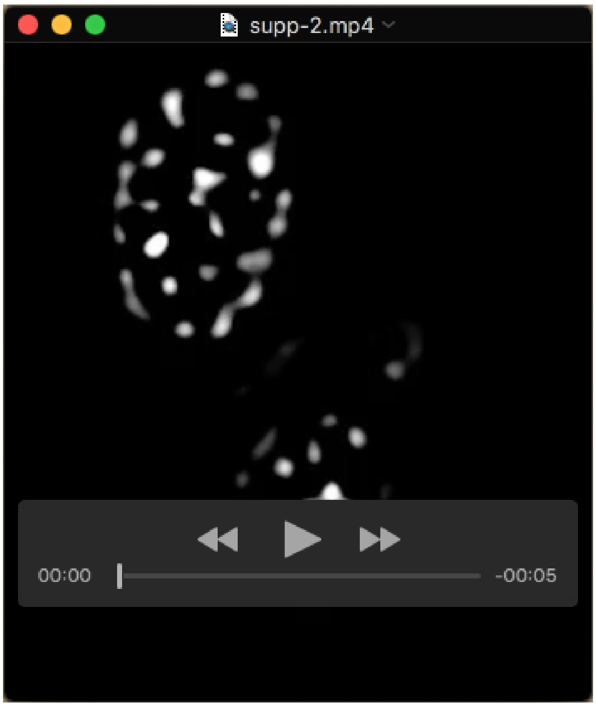

Movie 1. Animated Z-stacked images of Ssa1-DDR OSFs obtained by SIM microscopy.

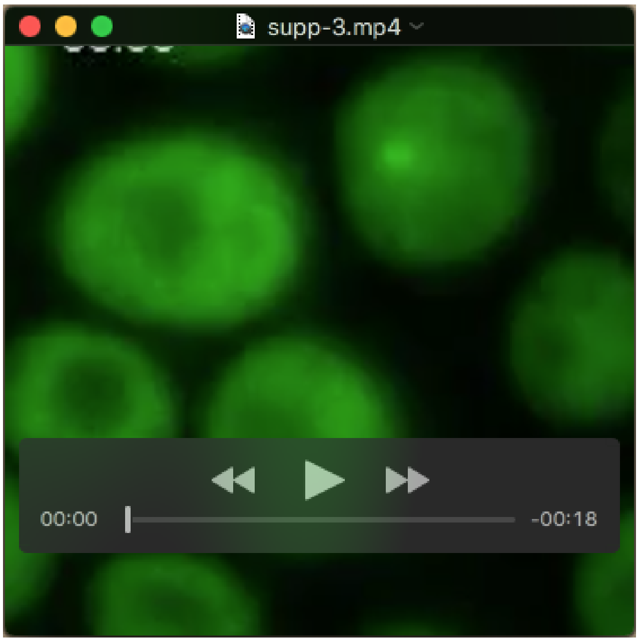

Movie 2. Video of sequential appearance and disappearance of OSFs in response to addition of KCl and its replacement for normosmotic medium. KCl added at 00:13, KCl exchanged for normosmotic medium at 01:59, new addition of KCl at 02:28.
